# Treasure of the Past IX: Exposure Standardization of Iodine-125 Seeds Used for Brachytherapy

**DOI:** 10.6028/jres.106.042

**Published:** 2001-10-01

**Authors:** T. P. Loftus

**Affiliations:** National Bureau of Standards, Gaithersburg, MD 20899

**Keywords:** calibration, exposure rate, free-air chamber, iodine-125 seed, re-entrant chamber, standards

## Abstract

A method for calibrating iodine-125 seeds in terms of exposure has been established. The standard free-air ionization chamber, used for measuring soft x rays, was chosen for the measurements. Arrays of four to six seeds were used to enhance the ionization-current-to-background-current ratio. Seeds from an array were measured individually in a re-entrant chamber. The quotient of the exposure rate for the array by the sum of the ionization currents in the re-entrant chamber is the calibration factor for the re-entrant chamber. Calibration factors were established for three types of iodine-125 seeds. The overall uncertainty for the seed exposure calibrations is less than 6%.

## Introduction

The radionuclide iodine-125, encapsulated in titanium seeds, is used for brachytherapy. Presently the seeds are characterized by stating a range for the activities of a group of seeds, with the stated activity computed from measurements made external to the seeds. In order to achieve traceability to NBS exposure standards, a manufacturer requested that NBS establish an iodine-125 calibration service for the seeds. Calibration data in terms of exposure for this radionuclide would then be consistent with calibrations for the radionuclides cobalt-60, cesium-137, and iridium-192. This procedure has also been requested by medical physicists and is in accord with recommendations of the National Council on Radiation Protection and Measurements [1].^1^

## Source Descriptions

**Figure 1:**
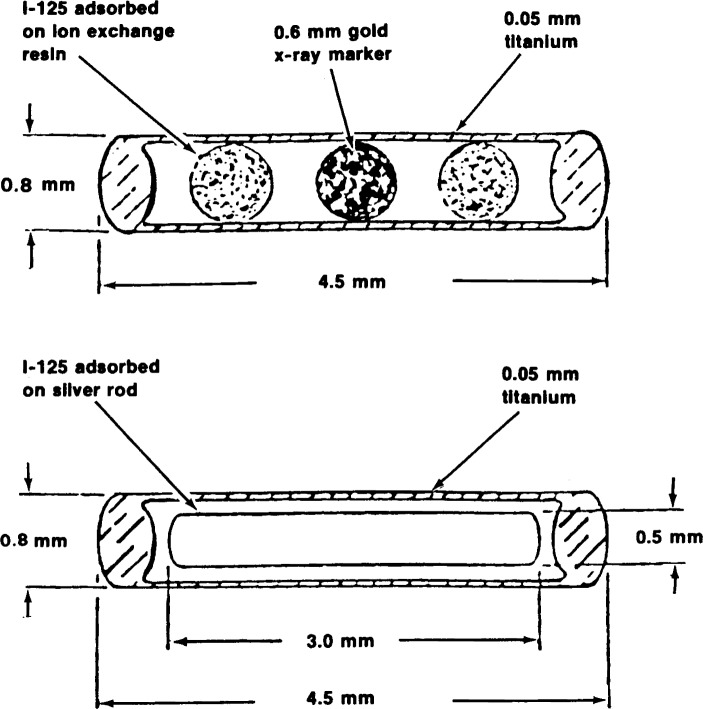
Cross section of gold-marker and silver-wire type iodine-125 seeds.

Three types of iodine-125 seeds were supplied for measurement. One type incorporates a gold marker which separates two resin spheres on which the radionuclide is adsorbed. The dimensions of the seed and of the spheres are shown in figure 1. The spheres are free to move in the seed as much as 1.5 mm with the result that the distribution of radiation near the end welds can change considerably.

A second type of source, also shown in figure 1, is a seed with the same dimensions as the gold-marker type, but with the radionuclide adsorbed on a silver wire 3.0 mm in length. The movement of the wire in the seed is restricted to 0.5 mm.

The third type of source, with higher activity than in the other two types, is one in which the gold marker sphere is replaced by another iodine-125-coated resin ball.

Mobility of the active material in the seeds does not appear to be an important factor in the primary standardization of the seeds, where good geometry measurements are made with a standard free-air ionization chamber, but movement of the active material affects the reproducibility of measurements in 4*π* geometry, as in the re-entrant ionization chamber used as a laboratory standard.

## Standard Free-Air Chamber: Measurement Conditions and Corrections

The instrument most nearly suitable for exposure measurements of radiation from iodine-125 is the Ritz [2] free-air ionization chamber (FAC). This is the standard chamber used for all NBS instrument calibrations for x rays in the 20-kV to 100-kV region. Important factors in the use of the FAC for the iodine-125 measurements are the defined air volume of the chamber (about 5.5 cm^3^) and the mean background current (about 1.6 fA). With these constraints, an iodine source of at least 400 MBq (12 mCi) would be required to provide measurement conditions of minimal acceptability, i.e., background corrections no greater than 10% of the readings and a source-to-chamber distance of no less than 0.25 m. The limit on improvement of the measurement conditions is governed by the available activity per seed and the physical size of the source relative to the FAC volume-defining aperture.

The important FAC dimensions, and corrections relevant to exposure measurements of iodine-125, are given in table 1. The photon-scattering and electron-loss corrections are those developed for measurements of 30-kV x rays filtered by 0.5 mm Al. There is no significant change in those corrections for x-ray energies down to 20 kV. The air attenuation correction given is based on measurements using the iodine-125 radiation. The magnitude of the air attenuation correction is in general agreement with corrections for x rays in the same energy region but is greater than the value calculated using mass attenuation coefficients [3] weighted for the source spectrum. The linear attenuation coefficient for air at room conditions based on the measurements is 1.5 × 10^−3^ cm^−1^. The calculated coefficient is 0.4×10^−3^ cm^−1^. In all subsequent computations, the measured coefficient was chosen to correct for air attenuation in the FAC and for attenuation between the sources and the defining plane of the FAC aperture.

## Source Arrays and Positioning Device

The maximum activity per seed (about 1.3 GBq, 36 mCi) is provided by the no-marker type seed (see table 2). To reduce the importance of background currents on the FAC measurements, the seeds were measured in groups.

Seeds of the same type were mounted on transparent tape and supported on a frame inside a thick-walled aluminum cylinder. They were affixed to the tape in a uniform array and the frame was positioned in the aluminum cylinder such that the array was in line with two diametrically opposite holes in the cylinder wall. The frame can be rotated while the center of the array is maintained at the center of rotation. The source array when in a vertical position is seen by the FAC as a source with dimensions 4.5 mm high and 0.8*n* mm wide, where *n* is the number of sources in the array. The number of seeds of each type ranged from four to six. The diameter of the FAC aperture is 10 mm, so these array dimensions do not present source-chamber alignment problems.

Two series of the three types of sources were measured on separate occasions. Information regarding the various groups is provided in table 2.

## Source Array Exposure Measurements

Although the source positioning device allows rotation of the source arrays, the main thrust of the FAC exposure measurements was to establish a mean exposure rate from an array of sources for the direction perpendicular to the plane of the array. This approach was taken since the eventual calibration of an individual seed will be given as a mean exposure rate at unit distance for the direction perpendicular to the long axis of the seed. To determine the mean exposure rate for a group of seeds, their positions and orientations were randomized on the transparent tape to make different arrays. The results of the first series of measurements for the gold-marker seeds are given in table 3.^2^ The mean for the three arrays at 0.50 m differs from the mean for the three arrays at 0.25 m by 1.5%, but the standard deviation of the mean for the 0.50 m data is 0.9%. Since different arrays were used (with one exception) for measurements at the two distances, the mean value for the exposure rate, for this group of seeds, is taken as the mean of the measurements for all the arrays. The mean for the arrays of gold-marker type sources in the first series is 2.336 μr m^2^/s on 1980 Dec 6.5. It is unimportant that array number 3 is included twice in computing the mean exposure rate.

All decay corrections for this work were computed using a half-life of 58.9 days for iodine-125. This half-life was determined when measurements of five seeds, taken 145 days apart and corrected using a half-life of 60.14 days (5), were all found to be inconsistent with earlier measurements. The average difference was 3.6%, with a standard deviation of the mean of 0.2%. The long-term stability for the measurement system used was shown, by 10 radium-226 check-source measurements, to have a standard deviation of the mean of 0.3%. Correcting the apparent activity and exposure data for decay, the exposure rate at unit distance per unit of apparent activity is 41.3 nR m^2^/“mCi”s.

## Calibration of a Re-Entrant Chamber for Routine Individual Source Measurements

A spherical aluminum re-entrant ionization chamber [4] was chosen as the means for transferring the FAC exposure data, for the mean exposure rate from an array, to exposure data for individual seeds. The original brass tube in the chamber was replaced with an aluminum tube with walls 0.64 mm thick. The new tube has an inside diameter slightly greater than the length of a seed and has a flat bottom. When the seed is dropped into the tube, it will lie horizontally and be constrained to take a position near the center.

The calibration of the re-entrant chamber consists in determining the quotient of the FAC exposure data, for an array of seeds, by the sum of the ionization currents produced in the chamber by individual seeds. The result is a calibration factor for the chamber in terms of exposure at unit distance per unit charge.

The mean exposures measured by the FAC are representative of exposure data for random seed orientations in the arrays. It is therefore necessary to randomize the seeds in the re-entrant tube to determine a mean ionization current for each seed. The variability of the currents with seed position is shown in table 4. The standard deviation of the mean for seed number 4 is a factor of 10 greater than the same statistic for measurements of an undisturbed seed.

The re-entrant chamber current measurements must be corrected for recombination. Tests show the correction factor is about 1.004 for currents of about 100 pA and unity for currents of about 0.8 pA. Interpolation for currents of about 20 pA (table 4) indicates the recombination correction is 1.001.

With the exposure data and re-entrant chamber currents corrected to the same date, the re-entrant chamber calibration factor for the first series of gold-marker type seeds is 3.842 kR m^2^/C.

### Iodine-125 Spectrum and Exposure Calculations

It is of interest to test the consistency of the iodine-125 exposure measurements with exposure data determined from other physical quantities. Such a check can be accomplished by comparison of exposure measurements with exposure data calculated from measurements of the iodine-125 spectrum, after attenuation in the seed wall, and decay data.

Iodine-125 decays by electron capture, producing tellurium x-rays, in addition to 0.0667 gamma rays per transition. Recent nuclear-decay data [5] are given in table 5, where energy-absorption data [3] are also listed. The equation used to compute the exposure rate at unit distance from a point source of unit activity in vacuum, is as follows:

$$\Gamma_{0}=\frac{\dot{X}L^{2}}{A}=\frac{1}{4\pi W/e}\sum_{i}{\left(PE\mu_{\text{en}}/\rho \right)}_{i}$$

where

*Ẋ*: is the exposure rate at a distance *L* from a point source in vacuum
*A*: is the activity of the source
*W/e*: is the mean energy expended per unit charge in air (33.7 J/C)
*P*: is the fraction of the nuclear transitions giving rise to photons of the associated energy
*E*: is the photon energy
*μ*_en_/*ρ*: is the mass energy-absorption coefficient for air for photons of the associated energy.

The exposure rate at unit distance per unit activity computed using the above equation and the data in table 5 is 42.2 nR m^2^/mCi s (after conversion to special units). The weighted mean uncertainty, for the value of *P* in table 5, is 1.8%, while the uncertainty given for the *μ*_en_/*ρ* data is 2% in this energy region. Obviously different values of Γ_0_ will result if different data for *P, E*, and *μ*_en_/*ρ* are used. A commonly used value is 40.3 nR m^2^/mCi s [6].

## Silver-Wire and No-Marker Type Seeds

The measurement procedures established for the gold-marker seeds were also used for the silver-wire and no-marker type seeds. Six arrays were measured for each type. The exposure data for the silver-wire type seeds are shown in table 6. These data are calculated from measurements at 0.25 m from the arrays. Table 7 gives the re-entrant chamber current measurements for each of the five silver-wire type seeds. The mean currents listed are the result of three independent sets of measurements, with each set the result of measurements for many randomized seed positions. With a correction for recombination of 1.004, the sum of the source currents is 562.4 pA. Correcting for decay to a consistent date and dividing the FAC exposure data by the sum of the re-entrant chamber currents gives a calibration factor of 4.467 kR m^2^/C for the first series of silver-wire type seeds.

The quotient of the exposure data, by the apparent activity given in table 2 for the same date is 40.64 nR m^2^/“mCi”s.

Exposure data for the first series no-marker type seeds are given in table 8. Six arrays of the four seeds, in randomized positions on the tape, were measured. The re-entrant chamber data are given in table 9 where the currents are corrected for recombination (1.004). The sum of these currents is 1.364 nA, and the quotient of the exposure data and re-entrant chamber data is 3.716 kR m^2^/C.

With corrections for decay, the quotient of the exposure data by the apparent activity given in table 2 is 38.30 nR m^2^/“mCi”s.

## Second Series, Au-Marker, Ag-Wire and No-Marker Iodine-125 Source Measurements

Completely independent sets of measurements for each of the three types of iodine-125 seeds were carried out to check the validity of the initial re-entrant chamber calibration factors. It was discovered that some scattered radiation from the cylindrical aluminum source container was being measured by the FAC and that a correction of 0.993 to the exposure data was required. At this time, the half-life of 58.9 days was introduced and recombination corrections for the re-entrant chamber were measured and used in the calculations. (All these improvements are incorporated in the data given for the first series of measurements.) For the second series of measurements, a lead plate 3 mm thick, and with an aperture matching the aperture in the aluminum cylinder, was used to eliminate the effect of scattered radiation on the FAC exposure measurements.

The measurement procedures established in the first series were for arrays of sources exclusively. In the second series, by using improved equipment it was possible to perform FAC measurements for single seeds as well. The data show that the FAC array measurements are not larger than the sum of the data for the individual seeds in the array. The data for the gold-marker seeds are given in table 10. The mean exposure rate, for the group of seeds, used to compute the re-entrant chamber calibration factor is the mean for all the measurements, i.e., the sums of the individual source measurements are averaged with the group measurements since there appears to be no significant statistical difference.

The results of many re-entrant chamber measurements of the gold-marker seeds are given in table 11.

The re-entrant chamber calibration factor is the quotient of the mean of the exposure data from table 10 by the sum of the re-entrant chamber currents corrected for recombination, i.e., 3.872 kR m^2^/C.

The data for the second series of measurements using the silver-wire sources are given in tables 12 and 13. The calibration factor for the re-entrant chamber computed from these data is the quotient of the mean for all FAC measurements by the sum of the mean re-entrant chamber currents corrected for recombination, or 4.400 kR m^2^/C.

The data for the second series of measurements for the no-marker type sources are given in tables 14 and 15. There is almost 2% difference between the single source sum data and the array data for exposure measurements. Since no significant difference for the two types of measurements was observed for the gold-marker and silver-wire sources, the large difference is believed to be random, and all data are included in the average for the exposure rate at unit distance. The quotient of the mean for the exposure data by the sum of the re-entrant chamber currents for the sources, corrected for recombination, is 3.701 kR m^2^/C.

## Summary of Calibration Factor Data for Re-Entrant Chamber

The calibration factors for the re-entrant chamber are summarized in table 16. The mean difference between the two independent sets of determinations of the calibration factors, referenced to their means, is 0.9%

## Comparison of Iodine-125 Source Calibrations: Exposure and Activity

A direct comparison of the exposure measurements and exposure data calculated from spectral measurements was made possible through the cooperation of the NBS Radioactivity Group. Two sources, one a gold-marker type and one a silver-wire type, were calibrated after decaying to activities within the range of radioactivity measurement equipment. The data are provided in tables 17 and 18 where *n* is the number of photons per unit time. The two sources were then calibrated for exposure using the re-entrant chamber and the calibration factors appropriate to their types. A comparison of the results is shown in table 19. It should be noted that although little self-scattered radiation was observed in the radioactivity measurement it is not counted, whereas the FAC with no discrimination will measure self-scattered radiation.

A problem associated with the use of activity as a measure for iodine-125 seeds is illustrated by the different values for this quantity which can be calculated from the spectrum for the gold-marker source (table 17) and decay data (table 5). Using *A = n/P* and the data for the 35.5-keV gamma ray, the computed activity is 2.5 MBq (69 μCi). If the data for the Te K*β* 31-keV x ray are used, the activity is 2.9 MBq (78 μCi); if the Te Kα data are used, the activity is 2.7 MBq (72 μCi). As a result, calculated gamma-ray exposure constants will differ depending on the reference radiation.

## Directional Dependence of Radiation from Iodine-125 Source Arrays

**Figure 2:**
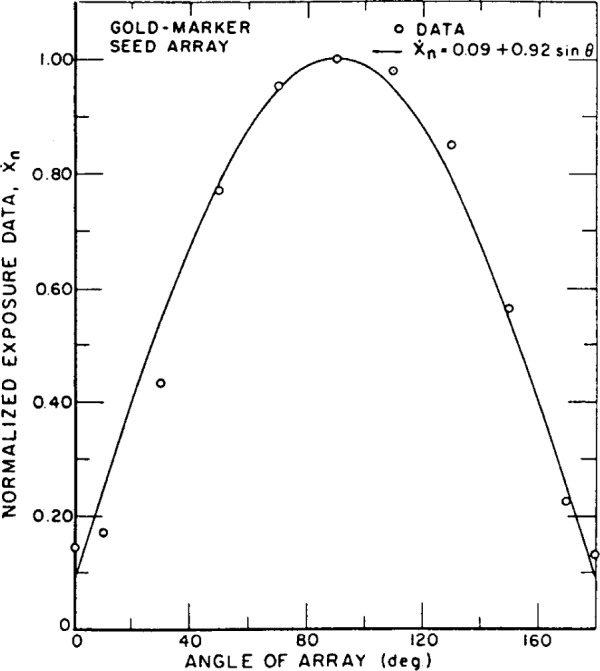
Standard free-air ionization chamber measurements of exposure rate from an array of gold-marker type iodine-125 seeds. The array was rotated through 180° starting and ending with the ends of the seeds facing the FAC diaphragm. The data are normalized to measurements with the plane of the array perpendicular to the FAC axis.

**Figure 3:**
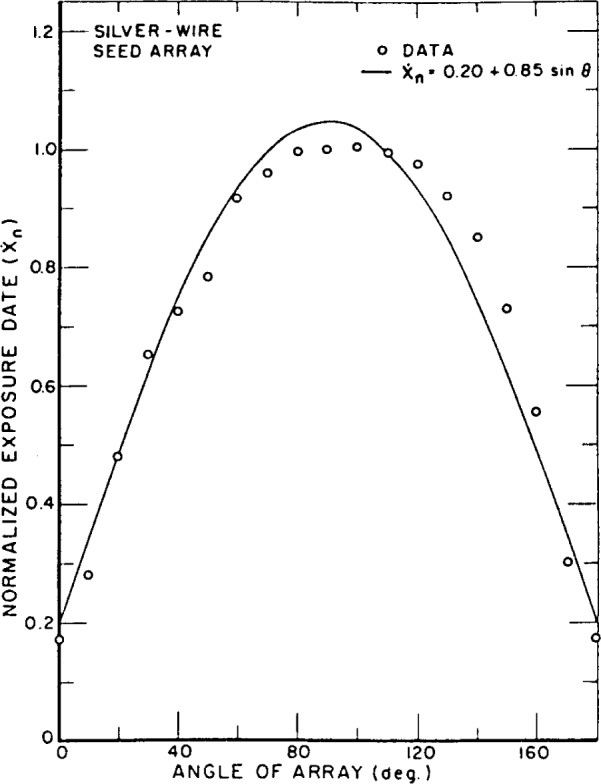
Standard free-air ionization chamber measurements of exposure rate from an array of silver-wire type iodine-125 seeds. The array was rotated through 180° starting and ending with the ends of the seeds facing the FAC diaphragm. The data are normalized to measurements with the plane of the array perpendicular to the FAC axis.

The calibration procedure for the iodine-125 seeds is designed to provide data for radiation emitted perpendicular to the seed axis. Since the seeds are implanted in tissue, the variation of exposure with direction from the source is of interest. Measurement of this characteristic was carried out by rotating the source arrays through 180° by means of the device already described. The exposure rate from a source array was found to be nearly a linear function of the sine of the azimuthal angle of the array (at 0 degrees, the source axes are parallel to the axis of the FAC). Normalized exposure data and *Ẋ_n_* = *f*(sin *θ*) are shown in figures 2 and 3. The data were normalized to unity at *θ* = *π*/2. The constants for the equations result from a least-squares fit to the data of a first order polynomial in sin *θ.* A mean exposure rate from a seed can be computed by averaging *f*(sin *θ*) over all solid angles so that

$${\bar{\dot{X}}}_{n}=\frac{\int_{0}^{\pi }{\dot{X}}_{n}\sin\theta d\theta }{\int_{0}^{\pi }\sin\theta d\theta }=a+\frac{\pi b}{4}.$$

The values for the constants and for ${\bar{\dot{X}}}_{n}$ are given in table 20. Higher order polynomials provide equations which fit the data better but the mean value for *Ẋ_n_* does not change when they are used. The mean exposure rates given in table 20 are for normalized data and are fractions of the exposure rates perpendicular to the long axis of the seeds. The results are in general agreement with the results of other authors [7,8].

## Uncertainties for Iodine-125 Exposure Standardization

The uncertainty for an exposure-rate calibration of an iodine-125 seed is dependent on the seed type. For each type, all random uncertainties, represented by standard deviations, and all remaining estimated uncertainties, which are treated like standard deviations, are added in quadrature to provide an estimate of the combined uncertainty. The random uncertainties in the determination of the re-entrant chamber calibration factor are the standard deviation of the mean for the exposure rate measurements of the group of sources, and the standard deviation of the mean for the total current in the reentrant chamber. These are given in table 21 for the first and second series of measurements. In this table, *n* is the number of sources in the array. The percentage given is the square root of the sums of the squares of the percent standard deviations of the means for each source. The standard deviation of the mean for each source is calculated from source measurements for random positions in the re-entrant tube.

The estimated uncertainties for the standard free-air chamber and re-entrant chamber are 1.2% [2] and 0.2%, respectively. The random uncertainties and the estimated uncertainties are added in quadrature to form a combined uncertainty. To the combined uncertainty for the re-entrant chamber calibration factor must be added the random uncertainty for the mean of several measurements of a seed in the re-entrant chamber during a calibration. This is estimated to be 0.4%.

The combined uncertainty is multiplied by 2 to give an overall uncertainty that corresponds roughly to a 95% confidence interval. The combined and overall uncertainties are given in table 22.

## Figures and Tables

**Table 1:** FAC dimensions, and corrections for measurement of iodine-125 radiation.

Diaphragm area	0.7867 cm^2^
Collector plate length	7.0003 cm
Air attenuation length	12.7 cm
Corrections:	
Air attenuation	1.018
Photon scattering	0.995
Electron loss	1.000
Recombination	1.000

**Table 2:** Iodine-125 seeds used for exposure measurements.

Type	Number of seeds	Apparent activity^1^ (“mCi”)	Assay date
	First series		
Gold-marker	4	60	1980/12/01
No marker	4	142	1982/09/06
Silver-wire	5	114	1981/06/01
	Second series		
Gold-marker	6	84	1983/02/21
No marker	4	103	1983/03/07
Silver-wire	6	95.4	1983/02/21

1 The apparent activity, as determined by the 3M Co., is a measure of the effective radiation output from the seeds and is independent of seed wall thickness or source self-absorption. The actual activity in the seed is greater than the apparent activity. The apparent activities are taken from the labels on the 3M lead acontainers. The 3M Co. has used the term “mCi Comp.” for apparent activity.

**Table 3:** First series exposure rate measurements for gold-marker type seeds (µR m^2^/s)

Array	FAC dist. (m)	Number of measurements^1^	Array means	Mean for arrays
1	0.50	4	2.281	
2	0.50	1	2.350	2.320
3	0.50	7	2.328	
3	0.25	5	2.350	
4	0.25	1	2.359	2.353
5	0.25	3	2.351	

1 Each measurement consists of an independent series of current measurements taken at different times.

**Table 4:** First series gold-marker type iodine-125 seed measurements in the re-entrant ionization chamber. Data corrected to STP and reference time 1981 June 1.5.

Source number	Mean current for random seed position (pA)	Number of seed positions	Standard deviation of the mean (%)
1	18.50	6	1.0
2	18.91	5	0.3
3	18.76	5	1.2
4	19.52	4	1.9

**Table 5:** Nuclear-decay and energy-absorption data used to calculate Γ_0_ for iodine-125.

Radionuclide	Radiation type	Energy *E* (keV)	Number per transition *P*	Energy-absorption coefficient *μ*_en_/*ρ* (m^2^/kg)	*E P μ*_en_/*ρ* (keV m^2^/kg)
Tellurium-125	x ray K*α*_2_	27.2	0.398	0.0212	0.230
Tellurium-125	x ray K*α*_1_	27.5	0.742	0.0204	0.416
Tellurium-125	x ray K*β*	31	0.258	0.0140	0.112
Iodine-125	*γ* ray	35.5	0.0667	0.0094	0.022

**Table 6:** First series exposure-rate measurements of Ag-wire type seeds measured at 0.25 m from the FAC (µR m^2^/s)

Array number	Number of measurements	Mean for 1981 June 1.5	Mean for all arrays	Standard deviation of the mean (%)
1	12	4.639		
2	5	4.593		
3	2	4.699	4.633	0.8
4	2	4.560		
5	2	4.762		
6	14	4.543		

**Table 7:** First series Ag-wire type iodine-125 seed measurements in the re-entrant ionization chamber. Data corrected to STP and reference time 1981 July 23.5.

Source number	Mean current (pA)	Standard deviation of the mean (%)
11	115.8	0.1
12	112.4	0.5
13	110.4	0.4
14	110.3	0.9
15	111.3	0.7

**Table 8:** First series exposure data for No-marker type seeds measured at 0.25 m from the FAC (µR m^2^/s).

Array number	Means for 1982 Sept. 12.5	Mean exposure rate (*µ*R m^2^/s)	Standard deviation of the mean (%)
1	5.040		
2	5.059		
3	5.060	5.068	0.2
4	5.070		
5	5.092		
6	5.086		

**Table 9:** First series No-marker type seed measurements in the re-entrant chamber. Data are corrected to STP and reference time 1982 Sept 12.5.

Source number	Mean current for seven measurements (pA)		Standard deviation of the mean (%)
1	338.8	0.2	
2	316.5	0.3	
3	338.4	0.4	
4	369.9	0.3	

**Table 10:** Second series gold-marker type iodine-125 source exposure measurements at 0.25 m from the FAC (μR m^2^/s).

Type of measurement	Number of measurements	Mean exposure data 1983 Feb 1.5	Standard deviation of the mean (%)
Single source, sum.	4	4.047	0.9
Arrays	5	4.054	0.7

**Table 11:** Re-entrant chamber current measurements for second series gold-marker iodine-125 seeds.

Source number	Number of measurements	Mean current 1983 Feb 1.5 (pA)	Standard deviation of the mean (%)
1	17	170.8	0.7
2	15	167.4	0.5
3	16	181.9	0.4
4	16	179.5	0.3
5	16	175.7	0.4
6	16	166.4	0.5
Sum 10.42 nA			

**Table 12:** Second series exposure measurements for silver-wire type iodine-125 sources at 0.25 m from the FAC (μR m^2^/s).

Type of measurement	Number of measurements	Mean exposure data 1983 Feb 1.5	Standard deviation of the mean (%)
Single source, sum.	5	5.613	1.3
arrays	8	5.688	1.0

**Table 13:** Re-entrant chamber current measurements for second series silver-wire sources.

Source number	Mean current 1983 Feb 1.5 (pA)	Number of measurements	Standard deviation of the mean (%)
1	208.6	6	0.2
2	219.5	6	0.5
3	195.0	6	0.2
4	228.9	7	0.7
5	230.4	11	0.8
6	198.7	6	0.9

**Table 14:** Second series exposure measurements for No-marker type iodine-125 sources at 0.25 m from the FAC (μR m^2^/s).

Type of measurement	Number of measurments	Mean exposure data 1983 Feb 1.5	Standard deviation of the mean (%)
Single source, sum.	3	5.490	0.3
Arrays	9	5.595	0.3

**Table 15:** Second series re-entrant chamber current measurements for the No-marker type sources.

Source number	Mean current^1^ 1983 Feb 1.5 (pA)	Standard deviation of the mean (%)
1	367.4	0.2
2	388.4	0.2
3	361.8	0.2
4	381.1	0.3

1 Currents for nine random source positions in re-entrant tube.

**Table 16:** Re-entrant chamber calibration factors (kR m^2^/C).

Source type	First series	Second series	Mean calibration factor
Gold-marker	3.842	3.872	3.857
Silver-wire	4.467	4.400	4.433
No-marker	3.716	3.701	3.708

**Table 17:** Measurements of gold-marker iodine-125 source spectrum (1983 Feb 21.44) and energy-absorption data.

Radiation type	*E* (keV)	*n* (s^−1^)	*μ*_en_/*ρ* (m^2^/kg)	*nE μ*_en_/*ρ* (keV m^2^/kg)
Te K*α*	27.4	2.402×10^6^	0.0210	1.382×10^6^
Te K*β*	31	5.867×10^5^	0.0140	2.546×10^5^
*γ*	35.5	1.340×10^5^	0.00935	4.448×10^4^

**Table 18:** Measurements of silver-wire iodine-125 source spectrum (1983 Feb 20.45) and energy-absorption data.

Radiation type	*E* (keV)	*n* (s^−1^)	*μ*_en_/*ρ* (m^2^/kg)	*nE μ*_en_/*ρ* (keV m^2^/kg)
Te K*α*	27.4	2.836×10^6^	0.0210	1632×10^6^
Te K*β*	31	7.028×10^5^	0.0140	3.050×10^5^
*γ*	35.5	1.962×10^5^	0.00935	6.512×10^4^
Ag K*α*	22.1	6.59×10^5^	0.0400	5.826×10^5^
Ag K*β*	25.2	1.61×10^5^	0.0268	1.087×10^5^

**Table 19:** Comparison of exposure calibration data, *Ẋ*(FAC) and exposure data computed from measurements of spectrum, *Ẋ*(*A*).

Source type	Exposure calibration (nR m^2^/s)	Exposure from spectrum (nR m^2^/s)	Ratio *Ẋ*(FAC)/*Ẋ*(A)
Gold-marker	3.271	3.117	1.049
Silver-wire	5.138	4.936	1.041

**Table 20:** Constants for the relation *Ẋ_n_=a+b* sin *θ*, and the normalized mean exposure rates for two seed types. The mean exposure rate is derived from data which were normalized to the exposure rate from the array in a direction perpendicular to the plane of the array.

Source array	*a*	*b*	${\bar{\dot{X}}}_{n}$
Silver-wire	0.20	0.85	0.87
Gold-marker	0.09	0.92	0.81

**Table 21:** Summary of standard deviations of the mean, *σ*_m_, for exposure and re-entrant chamber current summations.

	First series				Second series			

Source type	No. of arrays	*σ*_m_ FAC (%)	n	*σ*_m_ re-ent. (%)	No. of arrays	*σ*_m_ FAC (%)	n	*σ*_m_ re-ent. (%)
Gold-marker	5	0.6	4	2.5	9	0.5	6	1.8
Silver-wire	6	0.8	5	1.3	13	0.8	6	1.5
No-marker	6	0.2	4	0.6	12	0.3	4	0.2

**Table 22:** Combined and overall uncertainties for iodine-125 seed calibrations. The first and second series uncertainties are averaged to form the combined uncertainties.

Seed type	Combined (%)	Overall (%)
Gold-marker	2.6	5
Silver-wire	2.1	4
No-marker	1.5	3

## Footnotes

1 Figures in brackets indicate the literature references at the end of this paper.

2 The Si unit of exposure is the coulomb per kilogram (C/kg). This unit is not in general use, and the quantity exposure will probably be replaced by the quantity air kerma, which has the unit gray (Gy). As a result, the special unit roentgen (R) is used for exposure in this paper.
